# Morphological *vs*. molecular identification of trematode species infecting the edible cockle *Cerastoderma edule* across Europe

**DOI:** 10.1016/j.ijppaw.2024.101019

**Published:** 2024-11-14

**Authors:** Leslie Stout, Guillemine Daffe, Aurélie Chambouvet, Simão Correia, Sarah Culloty, Rosa Freitas, David Iglesias, K. Thomas Jensen, Sandra Joaquim, Sharon Lynch, Luisa Magalhães, Kate Mahony, Shelagh K. Malham, Domitilia Matias, Mélanie Rocroy, David W. Thieltges, Xavier de Montaudouin

**Affiliations:** a Univ. Bordeaux, CNRS, Bordeaux INP, EPOC, UMR, 5805, Station Marine d’Arcachon, Arcachon, France; b Univ. Bordeaux, CNRS, OASU, UAR, 2567, POREA, Pessac, France; c CNRS, UMR 7144 AD2M, Station Biologique de Roscoff, Sorbonne Université, Roscoff, France; d CESAM & Department of Biology, University of Aveiro, Campus Universitario de Santiago, 3810-193, Aveiro, Portugal; e School of Biological, Earth and Environmental Sciences and Aquaculture and Fisheries Development Centre, University College Cork, Cork, Ireland; f Environmental Research Institute, University College Cork, Cork, Ireland; g MaREI Centre, Environmental Research Institute, University College Cork, Cork, Ireland; h Centro de Investigacións Mariñas (CIMA), Consellería do Mar, Xunta de Galicia, Vilanova de Arousa, Spain; i Department of Biology, Ole Worms Allé 1, Building 1134, 8000, Aarhus C, Denmark; j Department of Sea and Marine Resources, Portuguese Institute for Sea and Atmosphere (IPMA, I.P.), Av. 5 de Outubro s/n, 8700-305, Olhão, Portugal; k Interdisciplinary Centre of Marine Environmental Research (CIIMAR), University of Porto, Terminal de Cruzeiros do Porto de Leixões, Av. General Norton de Matos s/n, 4450-208, Matosinhos, Portugal; l School of Ocean Sciences, Bangor University, Menai Bridge, Anglesey, LL59 5AB, United Kingdom; m GEMEL- Groupe d'étude des Milieux Estuariens et Littoraux, Saint-Valery-sur-Somme, France; n Department of Coastal Systems, NIOZ Royal Netherlands Institute for Sea Research, Den Burg, Texel, the Netherlands; o Groningen Institute for Evolutionary Life-Sciences (GELIFES), University of Groningen, Groningen, the Netherlands

**Keywords:** Molecular taxonomy, Trematodes, *Cerastoderma edule*, North-East Atlantic, cox1, SSU (18S) rRNA gene

## Abstract

Identifying marine trematode parasites in host tissue can be complicated when there is limited morphological differentiation between species infecting the same host species. This poses a challenge for regular surveys of the parasite communities in species of socio-economic and ecological importance. Our study focused on identifying digenean trematode species infecting the marine bivalve *Cerastoderma edule* across Europe by comparing morphological and molecular species identification methods. Cockles were sampled from ten locations to observe the trematode parasites under a stereomicroscope (morphological identification) and to isolate individuals for phylogenetic analyses using two gene markers, the small sub-unit ribosomal (18S) RNA gene (SSU rDNA) and the mitochondrial cytochrome *c* oxidase subunit 1 (cox1). For the first time, we compared both morphological identification and phylogenetic analyses for each of the 13 originally identified species. First, we identified a group of five species for which morphological identification matched molecular results (*Bucephalus minimus*, *Monorchis parvus*, *Renicola parvicaudatus*, *Psilostomum brevicolle*, *Himasthla interrupta*). Second, we identified a group of six species for which molecular results revealed either misidentifications or cryptic diversity (*Gymnophallus choledochus*, *Diphterostomum brusinae*, *Curtuteria arguinae*, *Himasthla quissetensis*, *H. elongata*, *H*. *continua*). Third, our analyses showed that all sequences of two expected species, *Gymnophallus minutus* and *G. fossarum*, matched between the two, strongly suggesting that only *G. minutus* is present in the studied area. Our study clearly demonstrates that molecular tools are necessary to validate the trematode species composition. However, with 17 distinct genetic lineages detected, some of which are not fully identified, future studies are needed to clarify the identity and status (regular *vs.* accidental infection) of some of these cryptic trematode species.

## Highlights

• Trematodes were identified in cockles from ten European locations.
• Half of the 13 optically identified species necessitate molecular confirmation.
• SSU and cox1 gene analyses suggested four supplementary genetic lineages.

## Graphical abstract

**Figure:**
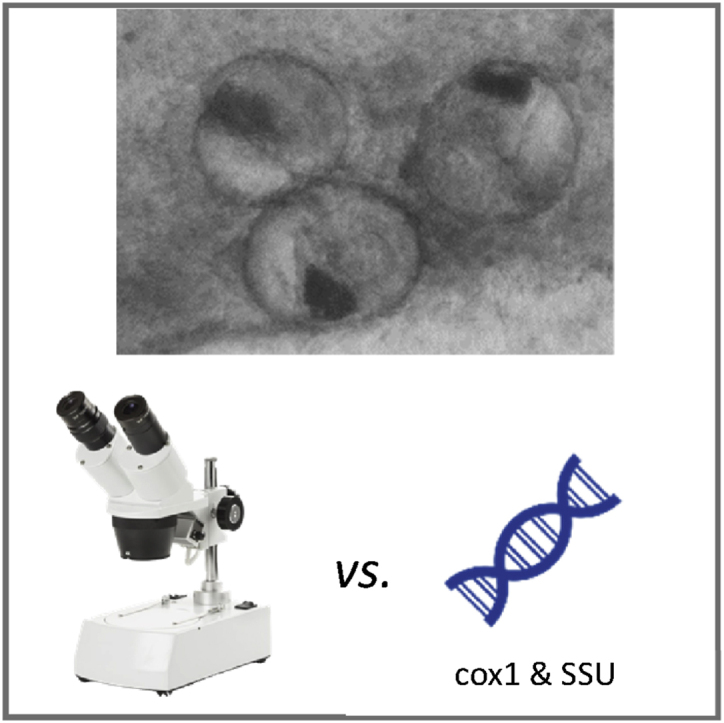

## 1 Introduction

**Fig. 1:**
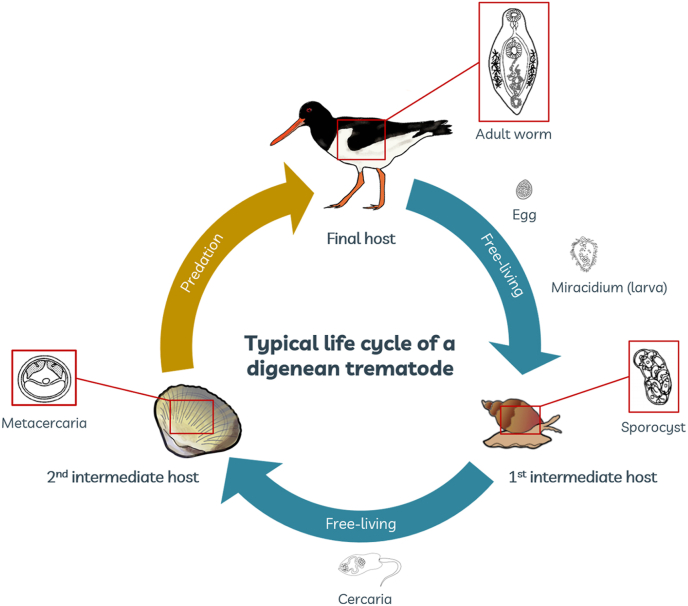
Example of a typical life-cycle of a digenean trematode using birds as final hosts.

Trematodes significantly contribute to marine biodiversity with well over 5000 marine species (Cribb et al., 2021), and are the most dominant group of macroparasites in intertidal ecosystems (Mouritsen and Poulin, 2002). They are characterized by having complex life-cycles that require two to four free-living hosts, most often three (Lauckner, 1983; Niewiadomska and Pojmańska, 2011) (Fig. 1). Due to their diverse host associations, digeneans are increasingly recognized as indicators for the presence of other taxa that are more difficult to study, acting as biodiversity proxies or potential indicators for environmental changes (Mackenzie, 1999; Hechinger and Lafferty, 2005; Duan et al., 2021; Stout et al., 2022; Moore et al., 2023). Furthermore, these parasites can have significant effects on their hosts at the individual level (Dairain et al., 2019), at the population level (Jensen and Mouritsen, 1992; Jonsson and André, 1992; Fredensborg et al., 2005; Thieltges, 2006) and in turn modify the structure of the overall community, inducing changes at the ecosystem level (Poulin, 1999; Dairain et al., 2019; Brian et al., 2022). In addition to their intrinsic ecological and biomonitoring value, it is even more crucial to monitor these parasites when they affect host species of particular ecological and economic importance (Lafferty and Hofmann, 2016).

Our study focuses on identifying the digenean trematode species that make up the parasite community infecting the edible cockle (*Cerastoderma edule,* Linnaeus, 1758). This bivalve is a common species in semi-sheltered intertidal and shallow subtidal systems along the northeastern Atlantic coasts, from the Barents Sea to Mauritania (Tebble, 1976; Honkoop et al., 2008). The edible cockle constitutes a crucial food source for birds, fish and benthic invertebrates (Malham et al., 2012). It provides numerous ecosystem services, such as biodiversity support, food production, erosion protection, and biochemical cycling (Carss et al., 2020). However, it is susceptible to various pathogens and diseases (Carballal et al., 2001; Longshaw and Malham, 2013; de Montaudouin et al., 2021), particularly digenean trematodes, which require careful monitoring. Thus, it is essential to accurately and efficiently identify parasite species down to the lowest taxonomic level. Traditionally, trematodes are identified morphologically under a stereomicroscope. Although histology can be used to detect trematodes, it is not suitable for species identification since only sections of the parasite are visible (Carballal et al., 2001). Some species are particularly difficult to detect due to their scarcity in tissues and/or their small size (miracidium larvae, early sporocyst stage). Even when detected, distinguishing them from other species can be difficult due to their indistinct morphological features. This is especially true when multiple species co-infect the same host, increasing the likelihood of missing cryptic species (McNamara et al., 2014). The use of molecular techniques for species determination is becoming increasingly common (Diaz et al., 2015; Miura et al., 2019; Schenk et al., 2020; Galaktionov et al., 2022; Bennett et al., 2023). DNA barcoding of single specimens using partial sequences of the mitochondrial DNA encoding for the cytochrome *c* oxidase I (cox1) gene has become standard practice. For trematodes, different gene markers are used. It is generally recommended to combine two separately evolving markers, a ribosomal marker (rDNA) and a mitochondrial marker (mtDNA) (Blasco-Costa et al., 2016). Barcoding has been used in previous studies to identify some of the trematodes parasitizing *C. edule* (*e.g.*; Francisco et al., 2010; Feis et al., 2015; Richard et al., 2022; Correia et al., 2023), but never the entire community as a whole. This has resulted in an incomplete molecular dataset with limited consistency in the genetic markers used.

The aims of our study were threefold. First, we aimed to compare species identification of the digenean trematode community in *C. edule* using morphological analysis versus single-specimen barcoding and phylogenetic analysis. Through this, we sought to determine the limitations of species identification via morphology alone and identify which species require molecular analyses as part of an integrative approach. Second, we aimed to investigate whether morphospecies across Europe belong to the same genetic lineages or if cryptic taxa are present. Finally, our third goal was to establish a reliable and uniform molecular database for nearly the entire digenean trematode community infecting *C. edule*. This database will serve as a reference for future barcoding efforts, with the ultimate goal of enhancing the accuracy of future monitoring of this parasite community.

## 2 Materials and methods

### 2.1 Cockle collection and examination

**Fig. 2:**
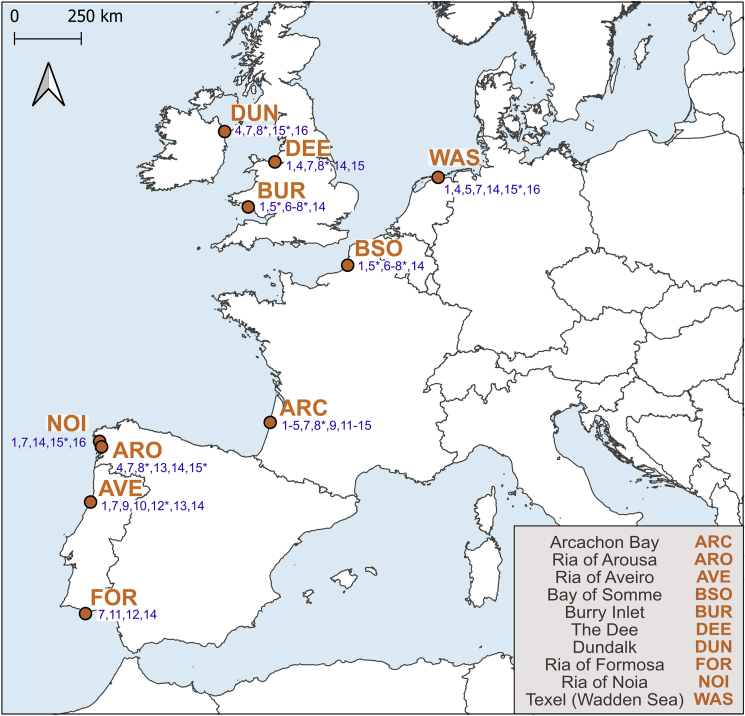
Map of the ten sampling locations along the European Atlantic coast. Numbers represent the trematode species/genetic lineages found infecting cockles in a given location, for which SSU and/or cox1 gene sequences were obtained during this study. 1: *Bucephalus minimus*, 2: *Monorchis parvus*, 3: *Renicola parvicaudatus*, 4: *Psilostomum brevicolle*, 5: *Gymnophallus choledochus*, 6: Gymnophallidae undet., 7: *Gymnophallus minutus*, 8: *Gymnophallus fossarum*, 9: *Diphterostomum brusinae*, 10: Zoogonidae undet., 11: Opecoelidae *s. l.* undet., 12: *Curtuteria arguinae*, 13: *Himasthla quissetensis*, 14: *Himasthla interrupta*, 15: *Himasthla continua* types 1–3, 16: *Himasthla elongata*. Asterisks indicate species that were incorrectly identified and are absent after molecular analyses.

Cockles were sampled at ten locations along the European Atlantic coast (Fig. 2) during two periods: pre-spawning (February–March) and post-spawning (September–November) (sampling details are available in Supplementary Table S1). During each sampling, 25 cockles per cohort were selected to increase the likelihood of finding infected individuals. The shell length of each cockle was measured with a caliper at the lesser mm (Supplementary Table S1). Taxonomic morphological identification of trematodes followed the procedure outlined by de Montaudouin et al. (2009), where the entire cockle tissue was squeezed between two thick glass slides and examined under a stereomicroscope and using transmitted light (Nikon, SMZ 1500). In addition to the cockles, we include in our analysis *Himasthla* cercariae shed by *Littorina littorea* (*H. elongata*) and *Peringia ulvae* (*H. continua*) from Denmark (sequences marked by “DAN∗“). Sporocysts, cercariae, and metacercariae, which had been previously identified at the species level, were individually isolated using forceps under a stereomicroscope. Each specimen was placed in a 1.5 ml tube and stored at −20 °C until further molecular analysis.

### 2.2 Molecular and phylogenetic analyses

#### 2.2.1 Molecular analyses

Genomic DNA was extracted from isolated sporocysts, cercariae, and metacercariae using the DNeasy Blood & Tissue Kit (Qiagen), following the manufacturer's instructions with a 3-h incubation for the lysis and tissue digestion phase. For all trematode species targeted in this study, we amplified two gene markers, the nuclear small sub-unit (18 S) ribosomal DNA (SSU) and the mitochondrial cytochrome *c* oxidase I DNA (cox1) (Table 1). We chose to use the SSU rDNA as our strategy was to combine the more variable cox1 mtDNA marker with a conservative rDNA marker. Though the large sub-unit (28 S) ribosomal DNA marker has gained in popularity for discriminating species and studying phylogenetic relationships, the SSU gene marker still proves to be useful for species discrimination and genetic diversity studies (Blakeslee et al., 2019; Wee et al., 2017). SSU sequence amplification was performed using previously published primers Bb18S and Bb18AS, which amplify ∼500 bp of the SSU-encoding gene (de Montaudouin et al., 2014). For cox1 sequence amplification, we designed new primers, TremCOIS2 and TremCOIAS2, first published by Magalhães et al. (2020), to increase the amplification success across all species in the studied community. These primers were developed based on a multiple sequence alignment of available cox1 sequences from closely related digenean trematode species, *Curtuteria australis* (KU748707), *Renicola sloanei* (KU563728) and *Maritrema novaezealandense* (GQ86823), aligned using Clustal W (v1.81, Thompson et al., 1994). Conserved regions suitable as candidate primer sequences were limited within the alignment, which resulted in an amplified sequence length of ∼250 bp. Cox1 amplification and sequencing was successful for almost all species, except for one, *Renicola parvicaudatus*.

**Table 1:** Primers used and their PCR amplification settings.

Target gene	Primer name	Sequence (5’→ 3′)	Amplified gene length	PCR cycling conditions	Reference
18 S	Bb18S	ACTGGAGGGCAAGTCTGGTGC	∼500 bp	94 °C/10min - (94 °C/60s - 59 °C/30s - 72 °C/90s) × 40 cycles - 72 °C/10min - 16 °C	de Montaudouin et al. (2014)
	Bb18AS	CAGCTTTGCAACCATACTTCCC			
COI	TremCOIS2	TGTTYTTTAGKTCTGTKAC	∼250 bp	95 °C/10min - (95 °C/60s - 43 °C/30s - 72 °C/60s) × 40 cycles - 72 °C/10min - 16 °C	Magalhães et al. (2020)
	TremCOIAS2	AATGCATMGGRAAAAAACA			

For every PCR reaction, a negative control (distilled H_2_O) was included. All PCR amplification reactions were performed in a 50 μl total volume using GoTaq G2 Flexi DNA polymerase (Promega, Madison, US), following the manufacturer's protocol (1X final concentration of Colorless GoTaq Reaction Buffer, 1.5 mM MgCl2 solution, 200 μM final concentration dNTPs, 1 μM of each primer, 5 U.μL−1 of GoTaq G2 Flexi DNA Polymerase and 1 μL template DNA). Cycling reactions for each primer set are detailed in Table 1. Amplified PCR products were checked on a 1% agarose gel stained with ethidium bromide. Positive PCR amplifications were sent for Sanger sequencing to Eurofins Genomics, Germany GmbH. Consensus sequences were assembled and edited using Geneious (https://www.geneious.com) and subsequently deposited in GenBank (accession numbers are provided in Supplementary Table S2).

#### 2.2.2 SSU and cox1 alignments and phylogenetic tree reconstruction

Using BLASTn, we recovered a broad sampling of SSU rDNA and cox1 mtDNA sequences, assembling 36 SSU and 16 cox1 published sequences into a multiple sequence alignment (Supplementary Table S2). As outgroup species, we included *Schistosoma incognitum* (AY157229)*, Schistosoma mansoni* (U65657 and NC002545)*, Schistosoma rodhaini* (AY157230), and *Schistosoma bovis* (OX103960). The sequences were aligned using MAFFT (v.7.487, Katoh et al., 2019) for the SSU alignment and MUSCLE (V5.1, Edgar, 2004) in MEGA 11 (Tamura et al., 2021) for the cox1 alignment. Both alignments were manually checked, resulting in a data matrix of 162 sequences and 554 alignment positions for SSU, and 110 sequences and 259 alignment positions for cox1.

Pairwise genetic divergence was determined using the simple-distance (*p*-distance) calculated in MEGA 11 using the default parameters (Supplementary Tables S3–4). Given the particularly difficult interpretation of the genetic variation within the *Himasthla continua* species complex, the distance-based Assemble Species by Automatic Partitioning (ASAP) (Puillandre et al., 2021) was used as a supplementary tool for species partitions within this group. The *p*-distance matrix based on the cox1 alignment of all sequences was uploaded at the website: https://bioinfo.mnhn.fr/abi/public/asap/. The partition with the lowest ASAP score was retained (Supplementary Fig. S1).

For the maximum likelihood (ML) phylogenetic analysis, the most appropriate nucleotide substitution model was selected using the model selection tool in IQ-TREE (1.6.12, Trifinopoulos et al., 2016), based on the Akaike information criterion (AIC). A sequence substitution model of GTR + F + I + G4 for SSU and TIM + F + I + G4 for cox1 alignments, each with four rate categories, was selected. The α parameter for the Gamma distributions 0.5184 for SSU and 0.718 for cox1. The ML phylogenetic tree was reconstructed in IQ-TREE using the previously identified parameters for each alignment. Nodal support was calculated with 10,000 nonparametric ultrafast bootstrap alignments (Hoang et al., 2018), using the same methodology. Additionally, Bayesian inferences (BI) were performed using MrBayes (v3.2.6, Ronquist et al., 2012) with the best-fit models GTR + I + G for SSU and TVM + I + G for cox1 (lset nst = 6, nucmodel = 4by4, rates = invgamma, ngammacat = 4, covarion = yes) based on the AIC from ModelGenerator (v0.851, Keane et al., 2006). Two independent Markov chain Monte Carlo chains (MCMC) were run for 2–4 million generations, respectively, with two replicate tree searches, each with 4 MCMC chains and a heat parameter of 2. Trees were sampled every 250 generations. The consensus topologies and posterior probabilities of each node were calculated from the remaining trees. The resulting posterior probabilities were mapped onto the ML phylogenies presented in this study using vector graphics softwares InkScape (1.2.2, retrieved from https://inkscape.org) and Affinity Designer 2 (v2.4.2, https://affinity.serif.com/fr/designer/).

## 3 Results and discussion

For 13 of the 16 digenean trematode species known to parasitize *Cerastoderma edule*, the sequence alignments of the SSU and cox1 gene markers included 36 and 16 publicly available sequences, respectively, along with 125 and 94 sequences recovered in this study (Supplementary Table S2). This study represents the first comparison of morphological identification and phylogenetic analyses for each of the 13 species. Three types of results were obtained: 1. Molecular results that confirmed the morphological identifications; 2. Molecular results that revealed misidentifications, which led to: 2.1. Improved discrimination of known or unknown species (cryptic diversity); and 2.2. The discovery that two morphospecies were actually the same in this investigation.

### 3.1 When morphological and molecular results match

The first type of results included a group of five species for which the molecular sequences of morphologically identified specimens clustered together as expected.

#### *3.1.1* Bucephalus minimus

**Fig. 3:**
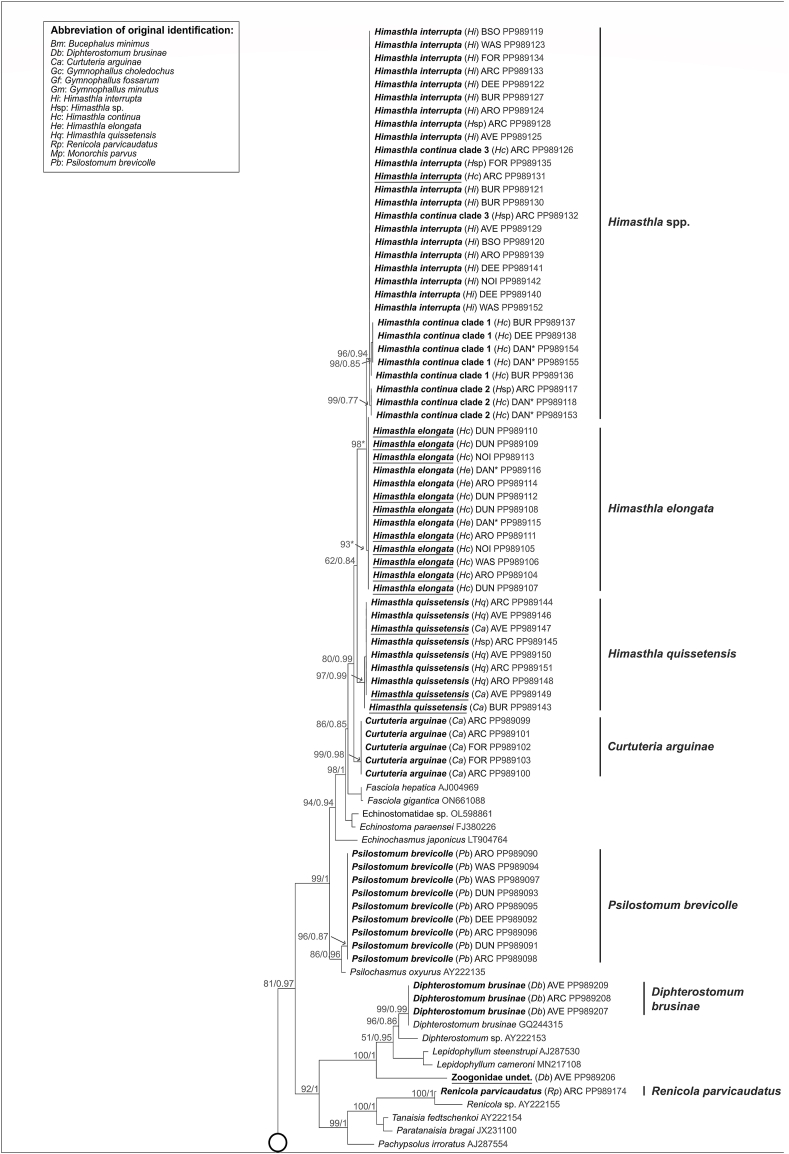

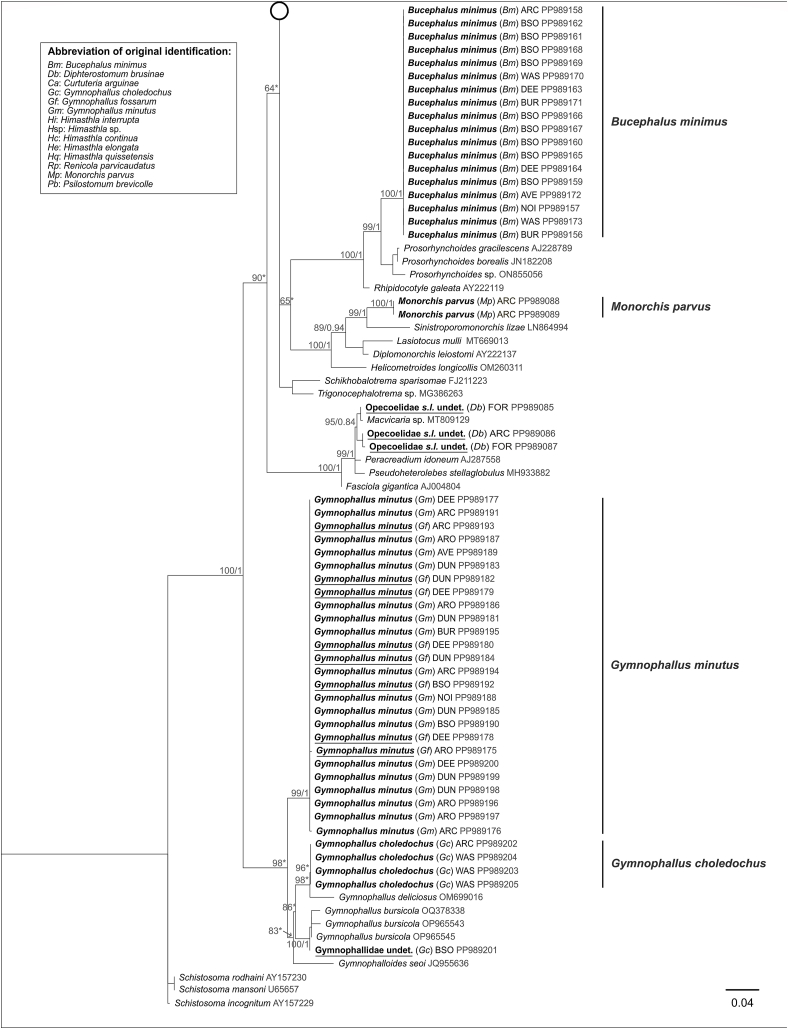
Phylogenetic relationships between 13 of the 16 digenean trematode species known to parasitize the cockle *Cerastoderma edule*, based on maximum-likelihood (ML) and Bayesian inference (BI) analyses of the partial SSU rRNA gene*.* The phylogeny is calculated from 162 sequences and 554 alignment positions. ML bootstrap support values followed by BI posterior probabilities were notated for relevant nodes. Asterisks indicate only bootstrap values, where BI resulted in a different tree topology. Sequences recovered from this study are represented in bold. The name of each of these sequences indicates as follows: the final species identification of the isolate after molecular analyses, abbreviation of the original morphological identification in parentheses, abbreviation of the sampling location (see Fig. 2) and GenBank accession number. Isolates which were originally incorrectly identified are underlined.

**Fig. 4:**
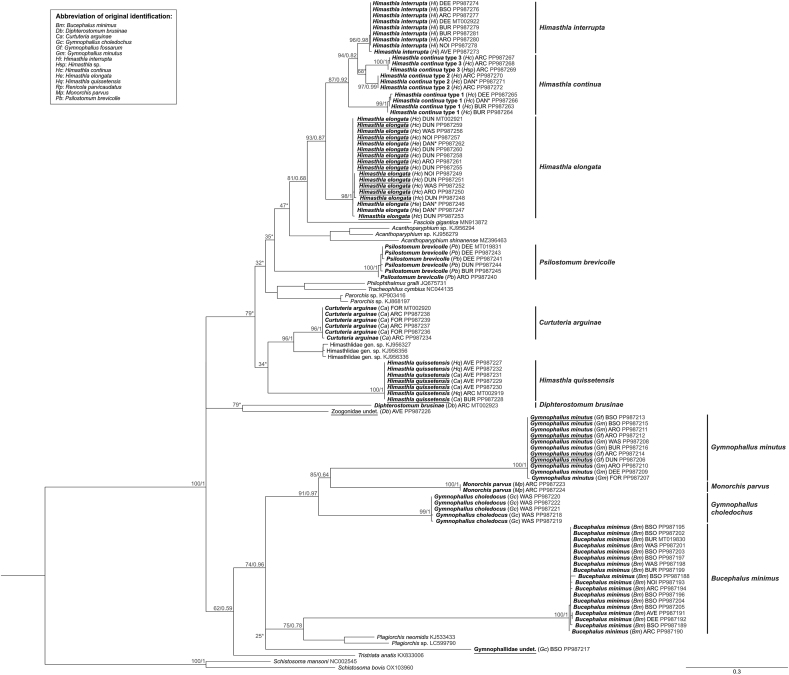
Phylogenetic relationships between 13 of the 16 digenean trematode species known to parasitize the cockle *Cerastoderma edule*, based on maximum-likelihood (ML) and Bayesian inference (BI) analysis of the partial cox1 gene. The phylogeny is calculated from 110 sequences and 259 alignment positions. ML bootstrap support values followed by BI posterior probabilities were notated for relevant nodes. Asterisks indicate only bootstrap values, where BI resulted in a different tree topology. Sequences recovered from this study are represented in bold. The name of each of these sequences indicates as follows: the final species identification of the isolate after molecular analyses, abbreviation of the original morphological identification in parentheses, abbreviation of the sampling location (see Fig. 2) and GenBank accession number. Isolates which were originally incorrectly identified are underlined.

*Bucephalus minimus* (Nicoll, 1914) was observed infecting cockles in the form of sporocysts and cercariae (Table 2), and identified according to Bartoli (1974). Eighteen partial SSU and cox1 sequences were recovered from sporocysts in cockles from seven locations (Fig. 2). The SSU sequences of *B. minimus* retrieved from cockles were identical and formed a highly supported clade (100/1), branching as a sister group of previously described species of Bucephalinae (Fig. 3). Although no published cox1 sequences associated with *B. minimus* covering the same gene fragment were available, the cox1 sequences retrieved in this study clustered together into a highly supported clade (100/1) (Fig. 4). These sequences exhibited minor polymorphism (0–1.6%).

**Table 2:** Digenean trematode species found infecting *Cerastoderma edule* in this study and their life-cycle. Host information was based on de Montaudouin et al. (2009).

	Family	Species	1st intermediate host	2nd intermediate host	Final host
**Cockles as 1**st **intermediate host**	Bucephalidae	*Bucephalus minimus*	*Cerastoderma edule*	*Pomatoschistus* spp.	*Dicentrarchus labrax*
				*Mugil cephalus*	
	Gymnophallidae	*Gymnophallus choledocus*	*C. edule*	*C. edule* or polychaetes	Water birds
	Monorchiidae	*Monorchis parvus*	*C. edule*	*C. edule*	*Diplodus* spp.

**Cockles as 2**nd **intermediate host**	Himasthlidae	*Himasthla quissetensis*	*Tritia reticulata*	*C. edule*	*Larus argentatus*
			*Tritia neritea*		
	Himasthlidae	*Himasthla interrupta*	*Peringia* spp.	*C. edule*	Laridae
	Himasthlidae	*Himasthla continua*	*Peringia* spp.	*C. edule*	Water birds
	Himasthlidae	*Himasthla elongata*	*Littorina littorea*	*C. edule*	Water birds
	Himasthlidae	*Curtuteria arguinae*	Unknown	*C. edule*	Unknown
	Gymnophallidae	*Gymnophallus minutus*	*Scrobicularia plana*	*C. edule*	*Haematopus ostralegus*
				*C. glaucum*	
	Gymnophallidae	*Gymnophallus fossarum*	*Scrobicularia plana*	*C. edule*	*Haematopus ostralegus*
				*C. glaucum*	
				*Polytapes aureus*	
	Psilostomidae	*Psilostomum brevicolle*	*Peringia ulvae*	*C. edule*	Laridae
	Zoogonidae	*Diphterostomum brusinae*	*Tritia reticulata*	*C. edule*	Fishes
	Renicolidae	*Renicola parvicaudatus*	*Littorina littorea*	*C. edule*	Laridae

The absence of misidentifications or cryptic genetic lineages within *B. minimus* across Europe, as revealed by our combined morphological and phylogenetic analyses, is consistent with previous studies in the same sampling area (Feis et al., 2015; Correia et al., 2023). The sporocysts and cercariae life stages, when associated to *C. edule,* are morphologically very distinct from those of other trematode species. Our results suggest that *B. minimus* can be identified morphologically under a stereomicroscope with high confidence.

#### *3.1.2* Monorchis parvus

*Monorchis parvus* (Looss, 1902) was observed infecting *C. edule* in the form of sporocysts containing cercariae or metacercariae, as this species uses cockles as its first and second intermediate hosts (Table 2). Identification was performed according to Bartoli et al. (2000). Two SSU and two cox1 sequences were recovered from sporocysts containing metacercariae in cockles from one location, Arcachon Bay (Fig. 2). The sequences were identical for each gene marker. The SSU sequences formed a highly supported clade (100/1), branching as a sister group to previously described species of Monorchiidae (Fig. 3). The identical cox1 sequences also formed a highly supported clade (100/1); however, no published cox1 sequences of monorchid species could be integrated into the analysis (Fig. 4).

Although the limited number of isolates retrieved in this study is insufficient to evaluate genetic variability within this species, recent studies highlight the autogenic nature of trematodes that exclusively use marine host species, suggesting a strong population genetic structure (Thieltges et al., 2009; Feis et al., 2015; Correia et al., 2023). Additionally, *M. parvus* has the particularity of typically developing from a cercaria to a metacercaria within the same host individual, which further reduces dispersal potential. Overall, the consistency between the morphological and molecular results support the notion that *M. parvus* can be accurately identified using the morphological approach.

#### *3.1.3* Renicola parvicaudatus

*Renicola parvicaudatus* (Galaktionov, Solovyeva, Blakeslee and Skírnisson, 2022) was observed infecting *C. edule* as metacercariae (Table 2), and was identified following Lauckner (1971). A unique SSU sequence was retrieved from a metacercaria encysted in a cockle from Arcachon Bay (Fig. 2), although no cox1 sequence could be obtained from this specimen. While this species was also found parasitizing cockles in three other locations (Fig. 2), no additional SSU nor cox1 sequences could be sequenced with sufficient quality. The cox1 primers we designed proved to be ineffective for this species. Nevertheless, the morphological identification of this specimen can be contextualized with molecular results integrating existing sequences retrieved from Genbank. Although recent molecular studies have produced a substantial number of genetic sequences of *R. parvicaudatus* (Galaktionov et al., 2022), these sequences could not be integrated into our analyses because they targeted different regions of the rDNA, specifically the large sub-unit (LSU) and the internal transcribed spacers (ITS) regions. However, the SSU *R. parvicaudatus* sequence that we retrieved formed a highly supported cluster (100/1) with a previously published sequence of the genus *Renicola*, specifically *Renicola* sp. (AY222155) (Fig. 3). This cluster was positioned as a sister group to three sequences from the Eucotylidae and Pachypsolidae, all placed in basal position. Previous studies have documented this close phylogenetic relationship between Renicolidae, Eucotylidae, and Pachypsolidae (Tkach, 2001; Olson et al., 2003). Thus, the molecular results strongly support the morphological identification of our isolate.

Indeed, morphologically, *R. parvicaudatus* presents distinctive features that minimize the likelihood of its confusion with other trematode species infecting cockles, including a thick cyst wall, dark secretory vesicles, and a specific tissue niche located in the palps. Galaktionov et al. (2022) reported minor intraspecific divergence between LSU and also cox1 gene sequences from *R. parvicaudatus* isolated from mollusks and gulls, but not from cockles. Therefore, additional sampling of *R. parvicaudatus* metacercariae infecting cockles is necessary to confirm that morphological identification carries minimal risk for error.

#### *3.1.4* Psilostomum brevicolle

*Psilostomum brevicolle* (Braun, 1902) was observed infecting *C. edule* as metacercariae (Table 2), and were identified based on the descriptions by Loos-Frank (1968) and Lauckner (1971). Nine SSU sequences and six cox1 sequences were generated from metacercariae encysted in cockles from five locations (Fig. 2). The SSU sequences were identical, forming a well-supported clade (96/0.87) branching as a sister group to the previously described psilostomid species within the larger Echinostomatoidea suborder (Fig. 3). Although no published cox 1 sequences were available, our cox1 sequences formed a highly supported clade (100/1) within Echinostomatoidea, with minor variability (0–1.1%) (Fig. 4).

These molecular results align perfectly with the morphological identifications, confirming that the species can be reliably identified under a stereomicroscope in cockles. Our findings also suggest that *P. brevicolle* may exhibit little intraspecific variation, despite being found in seven sampling locations across a broad geographic range in Europe. As an allogenic species using seagulls as its final hosts, this low genetic variability was expected (Feis et al., 2015).

#### *3.1.5* Himasthla interrupta

*Himasthla interrupta* (Loos-Frank, 1967) was observed as metacercariae encysted in the mantle margin of *C. edule* (Table 2) and were identified following the description by Lauckner (1971). Seventeen SSU and nine cox1 sequences were obtained from *H. interrupta* metacercariae collected from eight locations (Fig. 2). All SSU sequences were identical, yet the phylogenetic analysis did not provide the necessary resolution to group them in a distinct cluster within the Echinostomatoidea group (Fig. 3). The cox1 sequences, however, grouped into a single well-supported cluster (96/0.98), with all sequences showing identity except for one (PP987273), which exhibited a minor difference (0.5–0.8%) (Fig. 4). These molecular results confirmed the morphological identification, as observed previously by Richard et al. (2022), suggesting that the sampled specimens represent a single genetic lineage across Europe based on the SSU and cox1 gene markers. However, the SSU sequence cluster also encompassed three unidentified *Himasthla* species and two sequences identified morphologically as *H. continua*. Two of these sequences (PP989126, PP989132) did not cluster together with the *H. interrupta* sequences in the cox1 phylogenetic tree but instead formed a sister cluster representing a distinct clade, which we have designated as “*Himasthla continua* clade 3” (see section 3.2.5.2*.*).

No prior molecular work has been conducted on *H. interrupta*, limiting the ability to compare our results with existing findings. Similar to *Psilostomum brevicolle*, low intraspecific variability was expected for this widely distributed, allogenic species that uses seagulls as final hosts. The limited resolution (SSU) and length (cox1) of the markers used in this study could explain the lower-than-expected genetic variability observed.

In conclusion, five out of the thirteen trematode species parasitizing the cockle *C. edule* were accurately identified using a stereomicroscope examination. Notably, for the two species that utilize cockles as their first intermediate host (sporocyst and cercariae stages), identification is generally reliable, except during early stages of infection when visual cues may be less discernible. For species that use cockles as 2nd intermediate hosts only (metacercariae), identification was aided by either diagnostic traits (*P. brevicolle*), the infection of specific tissues (*H. interrupta*), or a combination of both factors (*R. parvicaudatus*).

### 3.2 When morphological and molecular data do not match

For an initial group of six species, the molecular sequences of morphologically identified specimens did not cluster as expected. The molecular results diverged from the morphological identifications revealing either partial misidentifications or the presence of cryptic diversity.

#### *3.2.1* Gymnophallus choledochus

*Gymnophallus choledochus* (Odhner, 1900) was observed as sporocysts containing cercariae in cockles (Table 2) and identified following Bartoli (1974). Five SSU and six cox1 sequences were obtained from sporocysts collected at three different locations (Fig. 2). Four SSU sequences were identical, forming a well-supported clade (96) in the ML analysis, while BI analysis resulted in a cluster including *G. deliciosus*. This clade branched as a sister group to previously described species from Gymnophallidae (Fig. 3). Similarly, though no published cox1 sequences could be included in our analyses, five cox1 sequences from different specimens formed a single, highly supported cluster (99/1), with minor polymorphism (0–0.8%) (Fig. 4). However, one sequenced specimen collected in the Bay of Somme (Fig. 2) differed, for which both SSU and cox1 sequences were generated (PP989201, PP987217). This isolate separated from the others but remained within Gymnophallidae in the SSU-based tree (Fig. 3). The SSU sequence diverged by 3.2% from the other four *G. choledochus* sequences, forming a highly supported cluster (100/1) with three *G. bursicola* sequences. In the cox1-based tree, this specimen formed a separate sister branch (no published *G. bursilicola* sequences were available) with 24% genetic divergence from the other *G. choledochus* sequences. Given these genetic differences, we revised this specimen's identification as an undetermined species of Gymnophallidae, subsequently labeled “Gymnophallidae undet.“). Overall, the morphological identification and molecular results for *G*. *choledochus* aligned well for most isolates, except for this one, which appear to have been misidentified as *G. choledocus*.

*Gymnophallus bursicola* is known to parasitize *Mytilus edulis* as its second intermediate host, though its first intermediate host remains unidentified (Benito et al., 2023). Gymnophallidae parasites typically use bivalves as first intermediate hosts. Considering the morphological similarities between the metacercariae of *G. bursicola* and *G. choledochus*, which may extent to the sporocyst stage, it was possible that this genetically distinct specimen was actually *G. bursicola*, despite this species has never been observed infecting *C. edule*. However, the genetic divergence based on 18 S between this sequence and the *G. bursicola* sequences is significantly greater than the intraspecific variation observed between *G. choledochus* sequences (3.2% > 0.80%). Therefore, we consider this hypothesis to be unlikely. A comparison using a less conserved marker, such as the cox1 gene, would provide more clarity, especially in light of a previous study (Feis et al., 2015) suggesting the potential existence of cryptic taxa within European *G. choledochus* populations.

#### *3.2.2* Diphterostomum brusinae

*Diphterostomum brusinae* (Stossich, 1904) uses cockles as its second intermediate host (Table 2). Identification of its metacercariae was based on Pina et al. (2009). Seven SSU sequences and two cox1 sequences were retrieved from metacercariae collected at three different locations (Fig. 2). The SSU sequences were distributed across three different positions in the phylogenetic tree (Fig. 3). Three SSU sequences from isolates collected in Aveiro (AVE) and Arcachon Bay (ARC) were identical and formed a highly supported clade (99/0.99) along with a previously published *D. brusinae* sequence (GQ244315), confirming their initial morphological identification (Fig. 3). This was partially corroborated by a single branch formed by one of the cox1 sequences (MT002923), which was a sister to another retrieved cox1 sequence (PP987226) (Fig. 4). However, the two sequences exhibited significant genetic divergence (19%), suggesting the presence of two distinct genetic lineages. The corresponding SSU sequence (PP989206), also formed a branch separate from the *D. brusinae* cluster, diverging by 7.9–13% from *D. brusinae* and *Diphterostomum* sp. (AY222153). Nevertheless, phylogenetic analyses placed the sequence within a highly supported cluster (100/1) exclusively composed of zoogonid species. The lack of intraspecific variability reported by Francisco et al. (2010) within *D. brusinae* from Aveiro suggests that the specimen most likely does not belong to the *Diphterostomum* genus but remains a member of Zoogonidae. As a result, we reclassified this specimen as an undetermined zoogonid species, subsequently named “Zoogonidae undet.“.

Additionally, three SSU sequences from two locations formed a separate well-supported cluster (95/0.84), together with a previously published sequence of the opecoelid *Macvicaria* sp. (MT809129) (Fig. 3). This cluster was positioned as a sister group to two other opecoelid species. These results suggest that the three corresponding specimens were misidentified as *D. brusinae* and potentially belong to another family, such as Opecoelidae.

Opecoelidae is the largest family of trematodes, comprising over 1000 species, many of which inhabit the Mediterranean and North-Atlantic waters. Opecoelid metacercariae, such as *Macvaria obovata*, can resemble *D. brusinae* metacercariae, with their large dark excretory vesicles (Born-Torrijos et al., 2012), making it possible to confuse these unexpected opecoelid metacercariae in *C. edule* for *D. brusinae*. This is particularly likely given previous reports of opecoelid trematodes in Arcachon Bay, where opecoelid sporocysts and cercariae were found infecting *Gibbula umbilicalis* (de Montaudouin et al., 2000). In Portugal, *Cainocreadium labracis* uses gobiid fish as its second intermediate host (Costa et al., 2016), ruling it out as a candidate species for our specimens. In the Mediterranean, the related gastropod *G. adansonii* serves as first intermediate host for two opecoelid species, *M. obovata* and *C. labracis* (Born-Torrijos et al., 2012). Given that *C. labracis* has been found in *G. umbilicalis* in the Atlantic, one might speculate that *M. obovata* could also be present*,* though it typically uses gastropods, not bivalves, as second intermediate hosts. Thus, while the metacercariae found in *C. edule* likely do not belong to these species, we cannot exclude the possibility that *C. edule* served as an accidental second intermediate host for an opecoelid species. In conclusion, no clear candidate species could be definitively identified as infecting cockles as its second intermediate host. Nonetheless, to our best knowledge, the three misidentified *D. brusinae* specimens likely belong to Opecoelidae and have thus been renamed as “Opecoelidae *sensu lato* undet.“. Further investigation is necessary to achieve a more precise identification by sequencing other regions of the rRNA gene array or other genetic markers.

#### *3.2.3* Curtuteria arguinae

*Curtuteria arguinae* (**Desclaux, Russell-Pinto, de Montaudouin and Bachelet, 2006)** was observed as metacercariae encysting in cockles, its second intermediate host (Table 2), specifically in the thin grey parts of the mantle and in the foot (Desclaux et al., 2006). Identification was performed following Desclaux et al. (2006). Eight SSU and ten cox1 sequences were retrieved from metacercariae collected from two southern locations (Fig. 2).

While no previously published *C. arguinae* sequences were available for inclusion in the analyses, five SSU sequences were identical and formed a highly supported cluster (99/0.98) within the larger of Echinostomatoidea clade (Fig. 3). Similarly, six identical cox1 sequences (with the exception of one containing an undetermined nucleotide) formed a well-supported branch (96/1), which was sister to three previously published Himasthlidae sequences isolated from marine snails in New Zealand (Keeney et al., 2015) (Fig. 4). Although these sequences were not identified to the species level, the authors noted that they matched most closely with *C. australis* sequences for both cox1 and ITS1 gene fragments. In the absence of molecular data of *C. arguinae*, these Himasthlidae sequences are the closest available taxonomic reference to support our findings. The proximity of these sequences to the *C. arguinae* group in the cox1 phylogenetic tree (Fig. 4) supports the accuracy of the morphological identification, further suggesting that the isolates belong to the *Curtuteria* genus (11% divergence from *C. arguinae*). However, other specimens morphologically identified as *C. arguinae* (3 SSU and 4 cox1 sequences) formed a separate, highly supported cluster (97/0.99 and 100/1 respectively) that included another species within the Echinostomatoidea, *Himasthla quissetensis,* for both gene markers. These specimens were thus revealed to be *H. quissetensis,* which had been incorrectly identified as *C. arguinae*, based on morphology.

These findings demonstrate that *C. arguinae* can be confused with *H. quissetensis* when both species infect the cockle's foot. However, *H. quissetensis* is always absent from the mantle, where *C. arguinae* can therefore be confidently identified. Despite general size differences, both species have a conspicuous excretory system, which can lead to confusion during identification. Though *C. arguinae* can be differentiated from *H. quissetensis* by the number of oral spines (33 in *C. arguinae* versus 31 in *H. quissetensis*, see de Montaudouin et al., 2009), suboptimal positioning of the excysted worm on a microscope slide can result in some spines being overlooked or counted twice (*e.g*., when broken), leading to misidentification. Our results underscore the importance of complementary molecular analyses to reliably differentiate *C. arguinae* from *H. quissetensis*.

#### *3.2.4* Himasthla quissetensis

*Himasthla quissetensis* (Stunkard, 1934) was found as metacercariae infecting the cockle's foot (Table 2) and was identified based on Stunkard (1938). The oral spines, often visible under a compound microscope, counted 31. As previously discussed, *H. quissetensis* may be confused with *Curtuteria arguinae* when both species infect the foot. Additionally, *H. quissetensis* could be challenging to distinguish from other congeneric species infecting cockles, especially *H. continua*, as their metacercariae are similar in shape, size, and foot location within cockles. The main distinguishing feature between the two species is the number of oral spines. However, when both species co-occur in the same host, the more conspicuous excretory system of *H. quissetensis* aids in identification. Five SSU sequences and three cox1 sequences were obtained from metacercariae morphologically identified as *H. quissetensis* from four locations (Fig. 2).

Though no previously published *H. quissetensis* sequences were available, all SSU sequences were identical, consistent with findings by Richard et al. (2022), and formed a highly supported cluster (97/0.99) within the broader Echinostomatoidea clade (Fig. 3). This cluster also contained sequences from a *Himasthla* sp. Isolate and three specimens morphologically identified as *C. arguinae*. Similarly, the cox1 sequences were identical (Richard et al., 2022), and, together with four identical sequences from specimens morphologically identified as *C. arguinae*, formed a highly supported cluster (100/1). This cluster was sister to three *Acanthoparyphium* sequences from Himasthlidae (Fig. 4).

As mentioned, *H. quissetensis* poses a high risk of misidentification. No misidentifications with *H. continua* were detected, contrary to expectations, but several specimens were wrongly identified as *C. arguinae*. This underscores the importance of carefully counting oral spines in challenging cases, or using complementary molecular analyses. Previous molecular studies revealed several genetically distinct lineages within specimens identified as *H. quissetensis* infecting the snail *Tritia obsoleta* in North America. Blakeslee et al. (2019) identified three clades based on the cox1 marker and two clades based on the SSU marker (not available in GenBank), indicating cryptic taxa within this species. In contrast, our study did not uncover cryptic taxa within *H. quissetensis*. This may suggest that individuals infecting cockles in Europe belong to a single clade, or that the sampling effort was insufficient to detect less common cryptic lineages.

#### *3.2.5* Himasthla spp.

##### *3.2.5.1* Himasthla elongata

*Himasthla elongata* (Dietz, 1909) and *H. continua* (Loos-Frank, 1967) were observed as metacercariae in cockles, (Table 2), and identified according to Lebour (1911) and Loos-Frank (1967). Both species share similar morphological characteristics, including the same number of spines (29) and the same microhabitat within the cockle (foot), making morphological distinction between the two particularly difficult, as noted by Richard et al. (2022). In total, 17 SSU and 21 cox1 sequences of morphologically identified *H. continua*, one SSU of *H. elongata,* as well as four SSU and one cox1 sequences of undetermined *Himasthla* sp. Metacercariae, were recovered from cockles sampled at seven locations (Fig. 2).

No previously published sequences were available for integration in our analyses (except for one SSU sequence of *H. elongata* that did not cover the same gene region). The sequences formed several clusters, which will be described successively.

Eleven SSU sequences (10 *H. continua* and one *H. elongata)* were identical and formed a well-supported cluster in the ML analysis (93∗) within the Echinostomatoidea (Fig. 3). The *H*. *continua* sequences recovered from cercariae shed by *Peringia ulvae* (1st intermediate host of *H. continua* and *H. interrupta*) collected in Denmark (marked “DAN∗“) were not part of this cluster. Instead, two sequences recovered from *H*. *elongata* cercariae shed by *Littorina littorea* (1st intermediate host of *H. elongata*) collected in Denmark (marked “DAN∗“) clustered within this group. Their sequences were identical with the others. Similarly, 14 cox1 sequences of *H. continua* formed a highly supported cluster (98/1) together with three sequences retrieved from *H. elongata* cercariae shed by *L. littorea* (Fig. 4). Cox1 sequences exhibited minor polymorphic variations in six out of 17 sequences (0.4–1.2%). Given that *H. elongata* is the only *Himasthla* species using *L. littorea* as its first intermediate host (Galaktionov et al., 2021), the morphological identification of cercariae released by this gastropod can be considered reliable. The molecular results reveal that all specimens morphologically identified as *H. continua* are, in fact, *H. elongata.*

##### *3.2.5.2* Himasthla continua clades

The remaining *Himasthla continua* and *Himasthla* sp. Sequences formed different clusters, although the results varied slightly between the two gene markers. Since the SSU gene marker is recognized as less resolutive than the cox1 gene (Moszczynska et al., 2009), we ultimately relied on the results based on the cox1 gene phylogeny for this complex. Accordingly, the remaining morphologically identified *H. continua* and *Himasthla* spp. Isolates encompass three distinct genetic clades that do not match those of other species (Fig. 4). The ASAP analysis based on the cox1 marker supports this partition into three distinct clades (Supplementary Fig. S1). As we do not know which cluster corresponds to the “real” *H. continua* (our expertise failed in finding morphological differences) for which no molecular data is available, these genetic clusters were consequently named “*Himasthla continua* clade 1”, “*Himasthla continua* clade 2” and “*Himasthla continua* clade 3”.

*Himasthla continua* clade 1 is represented by a well-supported cluster for both markers. On one hand, the SSU cluster (98/0.85) consisted of five *H*. *continua* sequences: three retrieved from cockles collected at two locations (Fig. 2) and two recovered from *H. continua* cercariae shed from *P. ulvae* from Denmark (marked “DAN∗) (Fig. 3). On the other hand, the three cox1 sequences recovered from metacercariae in cockles from two locations (Fig. 2), along with a sequence retrieved from a cercariae, formed the highly supported *H*. *continua* clade 1 (99/1) with minor genetic variation (0.4–1.2%) (Fig. 4).

*Himasthla continua* clade 2 is represented by an SSU cluster (99/0.77) consisting in one undetermined *Himasthla* sp. Metacercaria retrieved from a cockle from Arcachon Bay (Fig. 2) and two *H*. *continua* sequences recovered from cercariae (“DAN∗“), all of which were identical (Fig. 3). Three cox1 sequences, *i.e.* two sequences retrieved from metacercariae in cockles (Fig. 2) and one from a cercaria, were identical and formed a highly supported cluster (97/0.99), representing the *H. continua* clade 2 (Fig. 4).

*Himasthla continua* clade 3 was only detected through the cox1 gene. Three cox1 sequences recovered from metacercariae encysted in cockles from Arcachon Bay (Fig. 2) formed a highly supported cluster (100/1) with minimal genetic variation (0–0.4%) (Fig. 4). The cluster branched as a sister group to the *H. continua* clade 2, with 6.2–7.2% divergence. Two of the corresponding SSU sequences did not form a separate cluster but were identical to *H. interrupta* sequences. Three remaining SSU, initially identified as *Himasthla* sp. or *H. continua,* within this cluster were assigned to *H. interrupta,* as no cox1 sequences suggests otherwise (no cox1 sequence could be obtained for these specimens). However, it is possible that these specimens actually belong to the *H*. *continua* clade 3, like the other two sequences.

In conclusion, several trematode species infecting cockles cannot be definitively identified with certainty using a stereomicroscope. Sometimes, the confusion arises between different known species, such as *C. arguinae* and *H. quissetensis,* both of which are recognized parasites of cockles. In other cases, molecular results suggest the detection of new genetic lineages within a known genus, such as *Himasthla*, or even taxonomically distant species (*e.g*., from Zoogonidae and Opecoelidae). Supplementary molecular investigation is necessary for these complexes, using other genetic markers or a multi loci approach, combined with further morphological analyses to detect potential morphological differences and clarify the status of potentially cryptic species. At this stage, it is also impossible to determine whether the identified sequences belong to parasites specifically associated with cockles or if they correspond to accidental infection.

### 3.3 When molecular results decrease trematode diversity

Our third set of results showed the all sequences of two supposedly separated species, *Gymnophallus minutus* and *G. fossarum*, matched, strongly suggesting that the isolates actually correspond to the same species in this area.

#### *3.3.1* Gymnophallus minutus *and* G. fossarum

*Gymnophallus minutus* (Cobbold, 1859) was observed in *C. edule*, its second intermediate host (Table 2) as dark unencysted metacercariae in the mantle epithelium under the hinge. *G. fossarum* (Bartoli, 1965) metacercariae, similar in shape and size, were identified as free in the mantle margin tissues or extrapallial space. Identification followed Russell-Pinto (1990). We recovered 18 SSU sequences and seven cox1 sequences from morphologically identified *G. minutus* sampled in eight locations, and eight SSU and four cox1 sequences from morphologically identified *G. fossarum* sampled at five locations (Fig. 2).

In the SSU phylogenetic tree, all 26 sequences formed a highly supported cluster (99/1) (Fig. 3). No previously published sequences of either *G. minutus* nor *G. fossarum* could be integrated; however, the cluster formed a sister group to other *Gymnophallus* species. The cox1 sequences also formed a highly supported cluster (100/1) (Fig. 4).

Interestingly, sequences identified as *G. fossarum* were highly similar if not identical to the *G. minutus* sequences (0–1.6% genetic divergence). The differentiation between these species has been debated, as they share similar life cycles, infecting *Scrobicularia plana* as the first intermediate host and cockles (*Cerastoderma edule* and *C. glaucum*) as the second intermediate host, with oystercatchers *Haemotapus ostralegus* as the final host. Bartoli (1965, 1972) was the first to distinguish *G. fossarum* infecting lagoon cockles (*C. glaucum*), noting free metacercariae. He observed that morphological distinctions appear only in the adult stages (Bartoli, 1972). Bowers et al. (1990) experimentally infected *C. edule* with *G. fossarum* cercariae, resulting in free metacercariae and confirming the hypothesis of two allopatric sibling species: *G. fossarum* in *C. glaucum* in Mediterranean lagoons and *G. minutus* in *C. edule* along the Atlantic coast. Russel-Pinto first reported *G. fossarum* infecting *C. edule* in Portugal, where 57 % of *C. edule* were co-infected with enclosed and free metacercariae (Russell-Pinto, 1990; Russell-Pinto and Bartoli, 1992). Morphological measurements suggested significant size differences (Russell-Pinto, 1990), but a later study found no structural differences, concluding that differentiation requires ecological criteria (Russell-Pinto and Bowers, 1998). Here, for the first time, we compared molecular data of both species. Only two partial sequences of *G. minutus* are available in GenBank, and no molecular sequences of *G. fossarum* have been published, hindering comparison. Our results do not support the existence of two sympatric *Gymnophallus* species co-infecting cockles in the 10.13039/100004426Atlantic. Instead they suggest that all isolates belong to *G. minutus*, despite different microhabitats within the host. Indeed, no morphological differences were observed during the study. Free metacercariae co-occurring with enclosed metacercariae indicate unusual *G. minutus* metacercariae that have migrated to mantle margins. In Portugal, “*G. fossarum”* co-occurred with *G. minutus* but also singly infected *C. edule* in a few cases (Russel-Pinto and Bartoli, 1992). The presence of Mediterranean species in the Atlantic may be explained by overlapping oystercatcher populations, the final host of *G. fossarum*. However, this overlap is not known elsewhere along the Atlantic coast, where our study found one genetic lineage for both metacercariae types. This supports the hypothesis that both metacercariae in *C. edule* in the Atlantic are of the same species, *G. minutus.* Further molecular investigation of *G. fossarum* in the Mediterranean is needed for reliable classification of these similar *Gymnophallus* species.

### 3.4 Concluding remarks

A correct trematode monitoring program (*e.g.,* monthly intervals) requires reliable species identification and quantitative estimation (*e.g.,* number of metacercariae per species). Our study clearly demonstrated that molecular tools are necessary to validate the trematode species composition. However, future studies are needed to clarify the identity and status (regular *vs.* accidental infection) of some of these species. Based on SSU and cox1 gene markers, we detected 17 genetic lineages, with 15 potentially distinct species (compared to 13 expected by visual observation). Some of these could be accidental infections and/or reflect the accuracy limitations of the selected gene markers. On the other hand, such molecular analysis is currently unfeasible in terms of cost and time for recurrent surveys and cannot provide information on infection intensity. At this stage, our recommendation is to use molecular tools, for example, once a year to establish a list of possible taxa, and to maintain the classical “squeezing” technique (see section 2.) for higher-frequency sampling.

## CRediT authorship contribution statement

**Leslie Stout:** Writing – original draft, Visualization, Validation, Methodology, Formal analysis, Data curation, Conceptualization. **Guillemine Daffe:** Writing – review & editing, Validation, Methodology, Data curation, Conceptualization. **Aurélie Chambouvet:** Writing – review & editing, Validation, Supervision, Methodology, Formal analysis, Data curation. **Simão Correia:** Methodology. **Sarah Culloty:** Project administration, Methodology, Funding acquisition. **Rosa Freitas:** Project administration, Methodology, Funding acquisition. **David Iglesias:** Methodology. **K. Thomas Jensen:** Methodology. **Sandra Joaquim:** Methodology. **Sharon Lynch:** Methodology, Funding acquisition. **Luisa Magalhães:** Methodology. **Kate Mahony:** Methodology. **Shelagh K. Malham:** Project administration, Methodology, Funding acquisition. **Domitilia Matias:** Methodology. **Mélanie Rocroy:** Methodology. **David W. Thieltges:** Methodology. **Xavier de Montaudouin:** Writing – review & editing, Validation, Supervision, Project administration, Methodology, Funding acquisition, Formal analysis, Data curation, Conceptualization.

## Declarations of interest

None.
