# Supplementary material for: Morphological vs. molecular identification of trematode species infecting the edible cockle Cerastoderma edule across Europe
Source: Int J Parasitol Parasites Wildl. 2024 Nov 14;25:101019. doi: 10.1016/j.ijppaw.2024.101019 (PMC11648788; doi:10.1016/j.ijppaw.2024.101019)

Supplementary Fig. S1: Boxed subsets graph given by the ASAP analysis based on the cox1 alignment. The retained partition was the one with the lowest ASAP-score (column marked [1])


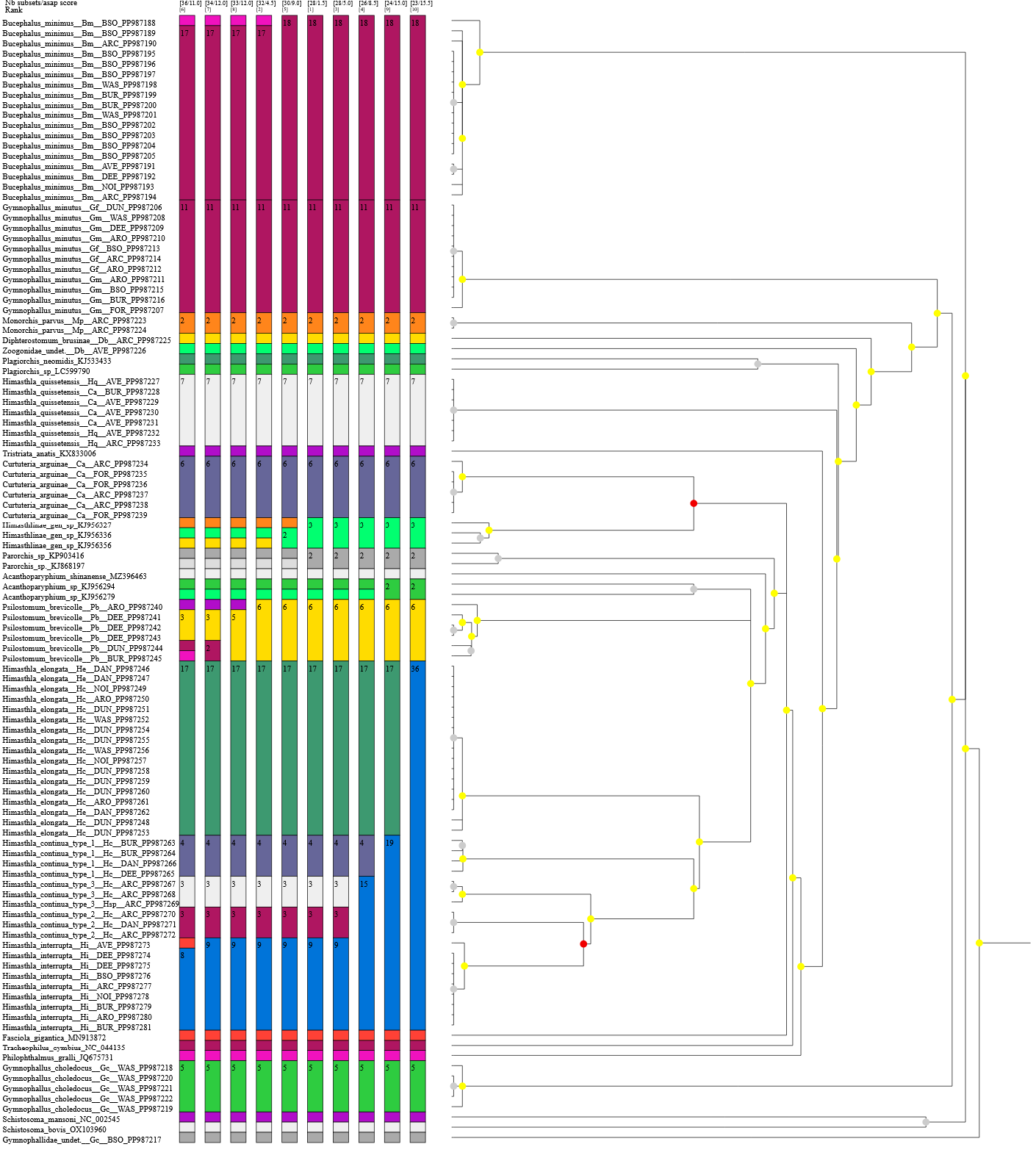

Supplement: Multimedia component 1 [file mmc1.zip › ijppaw_101019_mmc1/ijppaw_101019_STOUTE_spl_1_mmc1.DOC]
